# Formulation and Optimal Design of *Dioscorea bulbifera* and Honey-Loaded Gantrez^®^/Xyloglucan Hydrogel as Wound Healing Patches

**DOI:** 10.3390/pharmaceutics14061302

**Published:** 2022-06-19

**Authors:** Pattaranut Eakwaropas, Tanasait Ngawhirunpat, Theerasak Rojanarata, Prasopchai Patrojanasophon, Praneet Opanasopit, Nopparat Nuntharatanapong

**Affiliations:** Pharmaceutical Development of Green Innovations Group (PDGIG), Faculty of Pharmacy, Silpakorn University, Nakhon Pathom 73000, Thailand; eakwaropas_p@su.ac.th (P.E.); ngawhirunpat_t@su.ac.th (T.N.); rojanarata_t@su.ac.th (T.R.); patrojanasophon_p@su.ac.th (P.P.)

**Keywords:** xyloglucan, *Dioscorea bulbifera* extract, honey, hydrogel, wound dressing

## Abstract

Hydrogel patches are some of the most effective dressings for wound healing. In this study, the Gantrez^®^ S-97 (Gan)/xyloglucan (XG) hydrogel patches were formulated by using a full central composite design (CCD). The optimized hydrogel patches consisted of 17.78% *w*/*w* of Gan and 0.1% *w*/*w* of XG. Honey and *D. bulbifera* extract were loaded in the Gan/XG hydrogel patches. The physical properties of the hydrogel patches, including water content, water absorption, rate of water vapor transmission, and mechanical properties, were examined. The *D. bulbifera* extract/honey-loaded patch exhibited a higher value of water absorption, tensile strength, and elongation than the honey-loaded patch and the unloaded patch, respectively. The biological activities of the patches were also investigated. All hydrogel patches protected wounds from external bacterial infection. The *D. bulbifera* extract/honey-loaded patch exhibited stronger antioxidant activity than the honey-loaded patch and the unloaded patch. Besides, all the hydrogel patches with concentrations of 0.5–2.5 mg/mL showed that they were nontoxic to fibroblast cells. The combination of *D. bulbifera* extract and honey in the patch affected fibroblast proliferation. In addition, all Gan/XG hydrogel patches significantly induced recovery of the scratch area. Therefore, the Gan/XG hydrogel patches could be candidates as wound dressings.

## 1. Introduction

Hydrogels are the network structures of hydrophilic polymers’ crosslinking by physical or chemical methods. Several properties of hydrogels represent ideal dressings, such as hydrogel dressings could absorb wound exudate and preserve a moist environment of the wound area [1]. Nowadays, abundant hydrogel dressings are designed with the ideal dressing properties, for instance, cationic peptide-based salt-responsive antibacterial hydrogel dressings [2] and poly[3-(dimethyl(4-vinylbenzyl) ammonium) propyl sulfonate] (SVBA) in poly-acrylamide network hydrogel dressings (poly(AAm-co-SVBA) [3].

A number of polymers have been employed to prepare hydrogels for wound dressing. Gantrez^®^, a synthetic polymer of methylvinyl ether and maleic anhydride, has been used to develop pharmaceutical products for several purposes; for instance, Gantrez^®^ S-97 (Gan) and hyaluronic acid microneedles for transdermal delivery [4], Gan microneedles combined with lyophilized wafer for transdermal delivery [5], and Gan and hyaluronic acid microneedles for ocular delivery [6]. Recently, we successfully developed a thermal crosslinkable hydrogel-forming microneedle (MN) array using 30% *w*/*v* Gan as the MN-forming polymer [7]. Currently, there is no report for the use of Gan for wound-healing patches. Therefore, Gan was selected to formulate hydrogel wound dressings. Xyloglucan, a linear polysaccharide with side chains, functions as a storage polysaccharide in cell walls of higher plants and some tree seeds. Tamarind seed xyloglucan (XG) has been extensively studied and used as a thickener, gelling agent, starch modifier, emulsion stabilizer, ice-crystal stabilizer, etc., because of its wide-ranging stability regarding heat, salt, and pH [8]. It is thermally stable and could be degraded at temperatures between 300 and 370 °C. The glass transition temperature (T_g_) of XG is approximately 242 °C [9]. It is nontoxic and applied via several routes of drug delivery, such as buccal, nasal, ophthalmic, colon-targeted, and nasal routes [10,11,12]. Additionally, numerous studies reported the wound-healing stimulation of XG. It promoted skin renewal due to re-epithelialization and remodeling enhancement [13]. XG increased the corneal epithelium healing rate in albino rabbits [14]. Furthermore, the circular epidermal wounds treated with tamarind seed extracts exhibited shorter healing periods than the control in ICR mice [15]. Thus, XG has been developed and fabricated for wound dressings [16,17,18,19,20].

Nowadays, natural products are popular to be used as an active compound for wound healing. *Dioscorea bulbifera* is a traditional herbal medicine of the Dioscoreaceae family. It has been widely used to treat numerous diseases, including cancer, skin infections, coughs, abdominal pains, diabetes, leprosy, obesity, goiter, and inflammation [21,22,23]. Some studies reported on the wound-healing treatment of *D. bulbifera*. The tuber extract showed an improvement in wound contraction and a reduction of the epithelialization period compared with standard ointment [24]. Chaniad et al. found that four compounds from *D. bulbifera* bulbils significantly improved wound healing [25]. The authors also investigated viability, proliferation, and migration of fibroblasts treated with *D. bulbifera* extract gel for treating wounds. The results revealed that a concentration (lower than 500 mg/mL) of *D. bulbifera* gel affected fibroblast enlargement. Moreover, *D. bulbifera* gel produced a positively significant fibroblast cell migration [26]. Honey consists of monosaccharides, proteins, vitamins, minerals, flavonoids, polyphenols, and more. It has antimicrobial, antiviral, anti-inflammatory, antidepressant, and antioxidant properties [27,28]. Moreover, there have been reports on honey’s effect on wound healing in relation to human dermal fibroblasts. It was found that the jujube honey from Iran with concentrations of 100 and 200 µg/mL exhibited dose-dependent improvement in cell viability, proliferation, and migration [29]. Honey from stingless bees also exhibited a non-toxic effect on dermal fibroblasts, improved proliferation, and did not affect the normal cell cycle [30]. From these advantages, numerous studies were conducted on honey-loaded patches for wound dressings, such as pomegranate/honey PVA nanofibers [31], Manuka honey-loaded cellulose acetate nanofibers [32], and honey-loaded alginate/PVA nanofibers [33].

Therefore, in this study, XG was combined with Gan to prepare hydrogel dressings. The full central composite design (CCD) was used to determine the optimized film. Honey and *D. bulbifera* extract were loaded in the optimized hydrogels as active compounds for wound healing. The physical properties and biological activities of the hydrogel patches were investigated.

## 2. Materials and Methods

### 2.1. Materials

Gantrez^®^ S-97 (Gan) with MW of 1500 kDa was purchased from Ashland (Surrey, UK). Tamarind seed xyloglucan (XG) was purchased from Megazyme (Bray, Co. Wicklow, Ireland). *D. bulbifera* fresh bulbils were obtained from Uthong Hospital, Thailand. Honey was purchased from a local market in Thailand. 2,2-Diphenyl-1-picrylhydrazyl (DPPH) and 3-(4,5-Dimethyl-2-thiazolyl)-2,5-diphenyl-2H-tetrazolium bromide (MTT) were procured from Sigma-Aldrich (St. Louis, MO, USA). All other chemicals were of analytical grade. Dulbecco’s Modified Eagle’s Medium (DMEM) and supplements were purchased from Gibco BRL (Rockville, MD, USA). Normal human foreskin fibroblast (NHF) cells were obtained from the American-Type Culture Collection (ATCC, Rockville, MD, USA).

### 2.2. Preparation of D. bulbifera Extract

*D. bulbifera* fresh bulbils were cleaned, cut into small pieces, and dried in a hot-air oven at 50 °C. After grinding, 30 g of powder was macerated in 100 mL of 80% *v*/*v* ethanolic solvent at room temperature. Then, the extract was filtered and concentrated to achieve half volume.

### 2.3. Formulation of the Hydrogel Patches

Two water-soluble polymers: Gan with total concentrations of 15–18% *w*/*w* and XG with total concentrations between 0.10% and 0.25% *w*/*w,* were combined to fabricate the hydrogel patches by the thermal crosslinking method. First, Gan was dissolved in distilled water and stirred overnight. The XG was prepared by dissolving it in preheated distilled water at 50 °C while stirring for 4 h. The mixtures of Gan and XG solutions in various weight ratios, as shown in Table 1, were cast into the square molds and dried at room temperature for 3 days. Next, the dried films were peeled off and heated at 130 °C for 30 min followed by the thermal crosslinking method of Gan that was previously reported [7]. The crosslinked films were immersed in 50% *w*/*w* honey solution for 1 h.

### 2.4. Optimization of the Hydrogel Patches by Experimental Design

The response surface methodology coupled with the full CCD was used as a tool to design and optimize the hydrogel patches. The 2 independent factors (input), including A: Gan and B: XG, and the 6 responses (output), including Y_1_: water absorption, Y_2_: tensile strength, Y_3_: Young’s modulus, Y_4_: elongation, Y_5_: erosion, and Y_6_: water content, are shown in Table 1. A total of 13 experimental runs was statistically calculated by ANOVA with *p*-value < 0.05.

### 2.5. Embedding of D. bulbifera Extract to Optimized Film

The dried films were soaked in the 50% *w*/*w* honey and the mixture of *D. bulbifera* extract and honey with concentrations of 20% and 50% *w*/*w*, respectively. Then, the loaded hydrogel patches were taken out and the excess solution was removed using filter papers. The hydrogel patch without extract and honey was prepared as a control.

### 2.6. Fourier-Transform Infrared Spectroscopy (FT-IR)

The FT-IR spectra of Gan powder, XG powder, and dried films were recorded over wavenumbers ranging from 400 to 4000 cm^−1^ using attenuated total reflectance (ATR) (Nicolet iS5, Thermo Fisher Scientific, Waltham, MA, USA).

### 2.7. Water Content

The water contents of the loaded and unloaded hydrogel patches were examined. The patches were weighed and dried in a hot-air oven to achieve a constant weight. The water contents of the hydrogel patches were calculated from the weight difference between the initial weight and the constant weight after being heated, as per Equation (1):

(1) $$\mathrm{Water}\;\mathrm{content}\;\left(\%\right)\;=\;\frac{W_{i}-W_{d}}{W_{i}}\;\times \;100$$

where W_d_ and W_i_ are the weights of dried and initial patches, respectively.

### 2.8. Water Absorption

The swelling property of the hydrogel patch was investigated. The hydrogel samples were weighed and immersed in distilled water at 37 °C. After 24 h, excess water on the swollen sample surface was gently eliminated using filter paper before being weighed. The percentage of water absorption was calculated as per Equation (2):

(2) $$\mathrm{Water}\;\mathrm{absorption}\;\left(\%\right)\;=\;\frac{W_{s}-W_{i}}{W_{i}}\;\times \;100$$

where W_s_ and W_i_ are the weights of swollen and initial patches, respectively.

### 2.9. Erosion

The percent of erosion illustrates the crosslink strength of the hydrogel patches. The hydrogel samples were weighed and soaked in distilled water for 24 h. Then, they were heated at 60 °C until constant weights were obtained. The erosion was calculated as per Equation (3):

(3) $$\mathrm{Erosion}\;\left(\%\right)\;=\;\frac{W_{i}-W_{t\left(d\right)}}{W_{i}}\;\times \;100$$

where W_i_ and W_t(d)_ are the dried weights of initial patches and patches after water immersion, respectively.

### 2.10. Water Vapor Transmission Rate (WVTR)

To evaluate the WVTR of the optimized hydrogel patches, the patches were cut in a circular shape. The bottles that were filled with distilled water were mounted with the hydrogel patches. Then, the bottles were weighed (W_0_) and incubated at 37 °C, 75% RH, for 24 h. After incubation, the bottle weights were measured again (W_t_). The WVTR of the patch dressings was calculated from Equation (4):

(4) $$\mathrm{WVTR}\;=\;\frac{W_{0}-W_{t}}{A}$$

where A is the area of the bottle mouth.

### 2.11. Mechanical Properties

The tensile strength, elongation at break, and Young’s modulus of the hydrogel patches were measured using a texture analyzer. The hydrogel patches were cut into strips and fixed under a 5 kg load cell. The samples were stretched at a constant speed of 1 mm/s until the breaking point. The tensile strength, Young’s modulus, and the elongation at the patches’ breaking point were computed using Equations (5)–(7), respectively [34]:

(5) $$\mathrm{Tensile}\;\mathrm{strength}\;=\;\frac{\mathrm{Maximum}\;\mathrm{force}\;\mathrm{at}\;\mathrm{breaking}\;\mathrm{point}}{\mathrm{cross}\;\sec\mathrm{tion}\;\mathrm{area}}$$

(6) $$\mathrm{Young}’s\;\mathrm{modulus}\;=\;\frac{\mathrm{Stress}}{\mathrm{Strain}}$$

(7) $$\mathrm{Elongation}\;\left(\%\right)\;=\;\frac{\mathrm{Extension}\;\mathrm{of}\;\mathrm{length}\;\mathrm{at}\;\mathrm{breaking}\;\mathrm{point}}{\mathrm{Initial}\;\mathrm{length}}\;\times \;100$$

### 2.12. Infiltration Bacterial Test

The infiltration test was used to explain external bacterial protection of the hydrogel patch that was reported in the previous study [16]. In brief, the bacterial suspension was incubated at 37 °C overnight. Then, 10 μL of the bacterial suspensions was gently dropped in the center of the hydrogel patches that were placed on agar plates. The bacterial suspensions that were directly dropped on agar plates represented the positive control. After the agar plates were incubated at 37 °C overnight, three pieces of agar from three positions, including agar from the area under the patch, with bacteria, and distant from the patch (negative control), were incubated in broth at 37 °C. The bacterial growth was measured at various intervals using a spectrophotometer at a wavenumber of 600 nm.

### 2.13. Antioxidant Activity

Free radical scavenging activity of the hydrogel patch was assessed using the DPPH assay as previously reported [32,33]. To analyze the antioxidant efficacy of the hydrogel patches, each sample was weighted to 50 mg, soaked in 0.1 mM of DPPH ethanolic solution, and incubated for various periods at room temperature under dark conditions. The DPPH solution without patch dressings was regarded as a control. At different time intervals, the absorbance of the solution was measured at 520 nm using a UV-Vis spectrophotometer. The percent of antioxidant activity was calculated according to Equation (8):

(8) $$\mathrm{Antioxidant}\;\mathrm{activity}\;\left(\%\right)\;=\;\frac{\mathrm{Abs}.\;\mathrm{of}\;\mathrm{control}-\mathrm{Abs}.\;\mathrm{of}\;\mathrm{sample}}{\mathrm{Abs}.\;\mathrm{of}\;\mathrm{control}}\;\times \;100$$

### 2.14. NHF Cell Viability

The cytotoxicity of the hydrogel patch was investigated on NHF cells by indirect cytotoxicity and the MTT assay [33], with some alterations. To prepare the sample solutions, the hydrogel patches were exposed to UV light for 1 h on each side to be sterilized and then immersed in DMEM and shaken at 37 °C for 24 h to obtain extraction media. The NHF cells were seeded in 96-well plates at a density of 1 × 10^4^ cells per well and incubated at 37 °C, 5% CO_2_ atmosphere, for 24 h. After incubation, the culture medium was replaced with extraction media and further incubated for 24 h. Then, the extraction media were removed and the viability of NHF cells was evaluated using the MTT assay. Finally, the optical density at the wavelength of 550 nm was measured. The viability of NHF cells was calculated using Equation (9):

(9) $$\mathrm{Cell}\;\mathrm{viability}\;\left(\%\right)\;=\;\frac{\mathrm{Abs}.\;\mathrm{of}\;\mathrm{treated}\;\mathrm{group}}{\mathrm{Abs}.\;\mathrm{of}\;\mathrm{untreated}\;\mathrm{group}}\;\times \;100$$

### 2.15. NHF Cell Proliferation

For the cell proliferation assay, the extraction media were produced by soaking the hydrogel patches in the same culture medium as in the cell viability test. The NHF cells were seeded in a 96-well plate at a density of 5000 cells per well before being incubated with the extraction media for 24, 48, and 72 h. Then, the extraction media were discarded, and cell proliferation was investigated using the MTT assay. The percent of cell proliferation was calculated according to Equation (10):

(10) $$\mathrm{Cell}\;\mathrm{proliferation}\;\left(\%\right)\;=\;\frac{\mathrm{Abs}.\;\mathrm{of}\;\mathrm{treated}\;\mathrm{group}}{\mathrm{Abs}.\;\mathrm{of}\;\mathrm{untreated}\;\mathrm{group}}\;\times \;100$$

### 2.16. In Vitro Scratch Assay

The effect of the hydrogel patch on the migration of NHF cells was assessed by the in vitro scratch assay. The extraction media were prepared by immersing patches in the culture medium as previously described. Fibroblast cells were seeded into 6-well culture plates at a density of 2 × 10^5^ cells per well. The scratch area was generated on the NHF confluent monolayer using a micropipette tip. The detached area in each well was gently washed with PBS, pH 7.4. Thereafter, 3 mL of extraction media was added to each well, covering the cells. Images of the initial scratch areas and areas after their incubation for 24, 48, and 72 h were recorded under an inverted microscope (4×). The percentage recovery was calculated from the difference between the scratch areas before and after incubation as per Equation (11) [35]:

(11) $$\mathrm{Percentage}\;\mathrm{recovery}\;\left(\%\right)\;=\;\frac{A\;\left(t_{0}\right)-A\;\left(t\right)}{A\;\left(t_{0}\right)}\;\times \;100$$

where A(t_0_) is the wound area after immediate scratching and A(t) is the wound area after scratching at 24, 48, and 72 h.

### 2.17. Statistical Analysis

All measurements were collected in triplicate. The result values were represented as mean ± standard deviation (SD). All data were statistically analyzed using one-way analysis of variance (ANOVA) followed by Tukey’s post hoc test. The *p*-value < 0.05 was considered significant.

## 3. Results and Discussion

### 3.1. The Optimization of the Hydrogel Patches

The results of experimental runs and output responses are presented in Table 1. The experimental equation of hydrogel properties generated from the quadratic model was given as:

Y_i_ = b_0_ + b_1_A + b_2_B + b_12_AB + b_11_A^2^ + b_22_B^2^ (12)

where Y_i_ (i = 1 to 6) is the measured responses (hydrogel patch properties), including water absorption (Y_1_), tensile strength (Y_2_), Young’s modulus (Y_3_), elongation (Y_4_), erosion (Y_5_), and water content (Y_6_), respectively. The terms of A and B represent the experimental factors (formulation variables), including Gan and XG, respectively.

The relationship between independent factors and output responses is exhibited as coefficients of various terms in the polynomial equations (Table 2). A positive coefficient value expresses an increasing effect, whereas a negative one illustrates a decreasing effect on the hydrogel patch responses. The coefficient of determination (R^2^) is considered for the appropriate equation.

**Figure 1:**
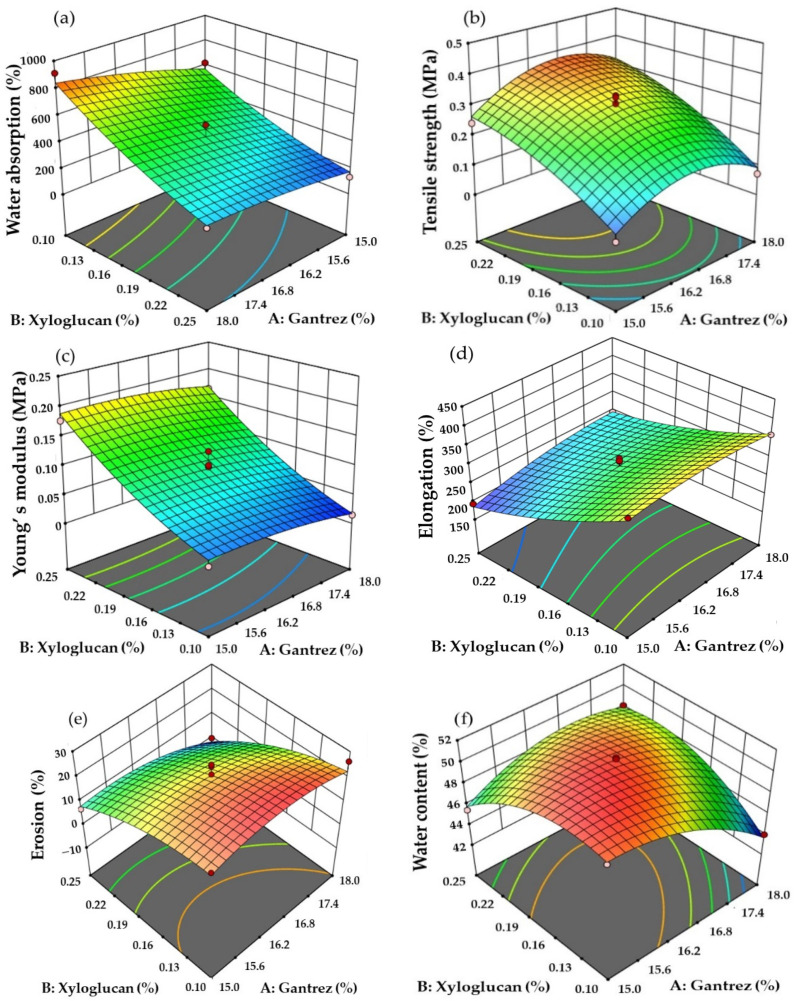
The 3D response surface plots of outputs: (**a**) water absorption, (**b**) tensile strength, (**c**) Young’s modulus, (**d**) elongation, (**e**) erosion, and (**f**) water content.

The three-dimensional response surface plots show that the amounts of Gan and XG significantly affected all output values (Figure 1). Water absorption values of the hydrogel patches were 130.93–912.42%. The water absorption of the hydrogel patches was affected by increasing the amount of Gan and decreasing the amount of XG (Figure 1a). The amounts of Gan and XG were related to the strength of the hydrogel patches (Figure 1b). The positive values of b_1_ and b_2_ showed that patch strength was enhanced when Gan and XG amounts increased. In addition, the amount of XG showed a more eminent effect on patch strength than Gan. The plot graph of Young’s modulus is shown in Figure 1c. The negative values of b_1_ showed a decreasing effect of Gan content on the Young’s modulus value, whereas the positive value of b_2_ indicated that Young’s modulus improved when XG content increased. Elongation values at the break point of the hydrogel patches were in the range of 194.10–420.15%. The polynomial equation and response surface plot for elongation are demonstrated in Table 2 and Figure 1d, respectively. The results indicated that elongation improved when Gan content increased, whereas XG content showed a decreasing effect on the elongation property. From the response surface plot in Figure 1e, it was found that both Gan and XG contents influenced the decreasing erosion. Moreover, XG content exhibited a more important effect than Gan. The effects of Gan and XG on water content of the hydrogel patches are shown in Figure 1f. Both Gan and XG indicated negative effects on water content.

**Figure 2:**
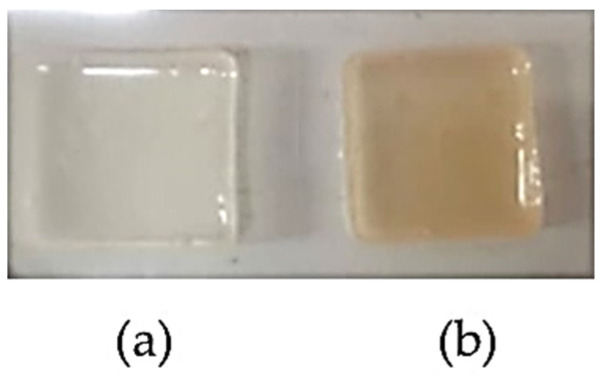
The optimized films (**a**) before and (**b**) after crosslinking at 130 °C for 30 min.

The aim of this experimental design was optimization of Gan and XG contents for wound dressing with criteria for maximum water absorption and maximum elongation. The optimization of the hydrogel patch formulation was 17.78% *w*/*w* of Gan and 0.1% *w*/*w* of XG. The predicted values of water absorption, tensile strength, Young’s modulus, elongation, erosion, and water content were 827.97%, 0.114 MPa, 0.021 MPa, 365.97%, 23.58%, and 44.68%, respectively. The optimized films which composed 17.78% *w*/*w* of Gan and 0.1% *w*/*w* of XG are shown in Figure 2. The thermal crosslinking led to a color change of the hydrogels without changing the dimensions and the size of the hydrogels (2.26 × 2.26 × 0.07 cm^3^). However, the weight of the hydrogels decreased by approximately 9.61% ± 1.40% due to the moisture evaporation during the crosslinking process. After the crosslinked films were immersed in 50% *w*/*w* of honey solution for 1 h, their physical properties were investigated. The results showed that water absorption, tensile strength, Young’s modulus, elongation, erosion, and water content were 824.20% ± 8.76%, 0.114 ± 0.005 MPa, 0.019 ± 0.003 MPa, 371.12% ± 10.60%, 21.23% ± 2.72%, and 44.80% ± 1.17%, respectively.

### 3.2. Embedding of D. bulbifera Extract to Optimized Film

For the extract loading, sodium hydroxide solution was added in the optimized mixture. After the mixture was cast into molds, dried, and crosslinked, the dried films were immersed in 50% *w*/*w* of honey and the mixture of *D. bulbifera* extract and honey with concentrations of 20% and 50% *w*/*w*, respectively.

**Figure 3:**
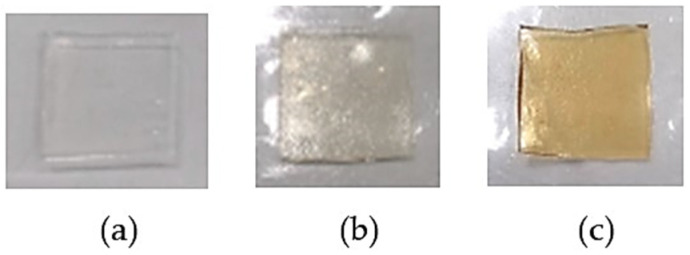
The hydrogel patches: (**a**) Gan/XG, (**b**) honey-loaded Gan/XG, and (**c**) *D. bulbifera* extract/honey-loaded Gan/XG.

The hydrogel patch which had been immersed in purified water was used as a control (unloaded hydrogel patch). The hydrogel patches loaded with 50% *w*/*w* of honey (honey-loaded Gan/XG), the mixture of *D. bulbifera* extract and honey with concentrations of 20% and 50% *w*/*w* (*D. bulbifera* extract/honey-loaded Gan/XG), and the unloaded hydrogel patch (Gan/XG) are shown in Figure 3. The Gan/XG hydrogel patch was transparent and colorless. Loading of honey and *D. bulbifera* extract into the crosslinked hydrogels by absorption led to the change in the color of the hydrogels to light brown and light orange colors due to the color of honey and the extract. As compared to the hydrogels loaded with water, the hydrogels loaded with honey alone and the honey/extract mixture exhibited a somewhat lower swelling due to the higher viscosity of the media, resulting in a lower penetration into the hydrogels compared to water. This led to a slight change in the dimension and the weight of the hydrogels. The size of the hydrogel patches was 3.05 × 3.22 × 1.08 cm^3^ after water absorption, whereas the size was 3.02 × 3.18 × 1.04 cm^3^ and 3.01 × 3.16 × 1.02 cm^3^, respectively, for the hydrogels absorbed with honey and the honey/extract mixture. The weight of the hydrogels increased by 792.11% ± 9.49% after immersion in the water, while the weight change of the hydrogels was 778.38% ± 7.15% and 754.05% ± 5.41% after immersion in honey and the *D. bulbifera* extract/honey mixture.

### 3.3. FT-IR Spectra

**Figure 4:**
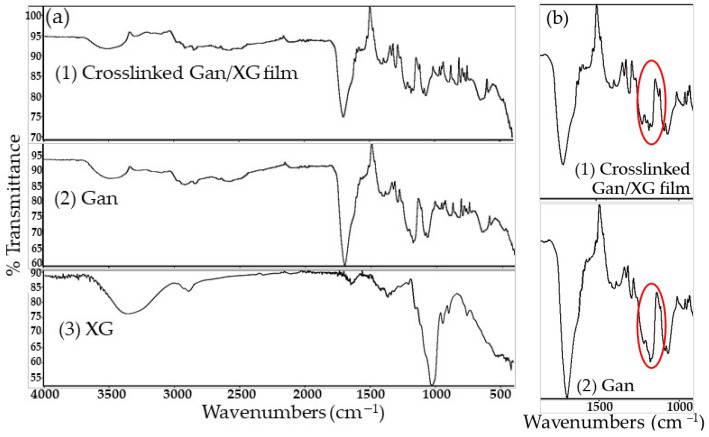
(**a**) The FT-IR spectra of (1) the crosslinked Gan/XG film, (2) Gan powder, and (3) XG powder. (**b**) Expanded FT-IR spectra of (1) the crosslinked Gan/XG film and (2) Gan powder. Red circles show the new spectra of the crosslinked Gan/XG film compared with Gan powder.

The Gan/XG film crosslinking was confirmed by FT-IR. Figure 4 shows the FT-IR spectra of XG powder, Gan powder, and the crosslinked Gan/XG film. The new spectra of the crosslinked Gan/XG film occurred at approximately 1100–1300 cm^−1^ (in the red circle) after the thermal crosslinking process, which represented the bands of the C-O stretching bond.

### 3.4. Water Content

Water contents of the hydrogel patches are shown in Table 3. The water content of honey-loaded Gan/XG was nearly the same as *D. bulbifera* extract/honey-loaded Gan/XG, whereas the unloaded hydrogel patch had much more water content than the others.

### 3.5. Water Absorption

Water absorption of the loaded and unloaded hydrogel patches is shown in Table 3. All hydrogel patches could absorb high amounts of water due to the super swelling property of Gan, as previously reported [5]. Both the loaded hydrogel patches exhibited significantly higher water absorption compared with the unloaded hydrogel patch. Moreover, water absorption of *D. bulbifera* extract/honey-loaded Gan/XG was significantly higher than honey-loaded Gan/XG.

### 3.6. WVTR

Transmissibility of water vapor through the hydrogel patches is shown in Table 3. Water vapor transmission rates of both the loaded hydrogel patches were 516.51 and 576.62 g/m^2^/day, which correlated to the semi-permeable film dressing property [36]. The WVTR of the unloaded hydrogel patch could not be observed. The unloaded patch was not able to completely cover the bottle mouth due to high water evaporation from the patch that affected the patch contraction.

### 3.7. Mechanical Properties

The strength, stiffness, and flexibility of the hydrogel patches were evaluated by the tensile test. Young’s modulus, tensile strength, and percent of elongation were observed (Table 4).

Honey incorporated in the Gan/XG patch significantly increased Young’s modulus, tensile strength, and elongation compared with the unloaded Gan/XG patch, expressing greater stiffness, strength, and extensibility. The results of the *D. bulbifera* extract/honey-loaded hydrogel patch demonstrated that the combination of extract and honey had significantly higher tensile strength and elongation than the unloaded patch, which explained the higher strength and elasticity of the patch. Furthermore, *D. bulbifera* extract and honey loading enhanced the extensibility of the patch more than the honey loading only. The addition of *D. bulbifera* extract could promote the elasticity of the patch more than honey alone.

### 3.8. Infiltration Bacterial Test

**Figure 5:**
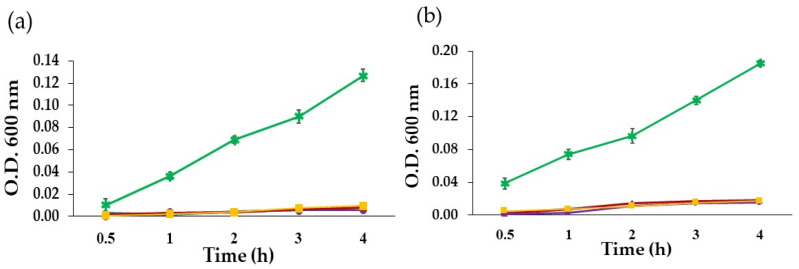
Infiltration bacterial test: (**a**) *S. aureus* and (**b**) *E. coli* of hydrogel patches: (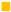) agar without bacteria, (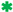) agar with bacteria, (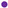) Gan/XG hydrogel patch, (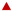) honey-loaded Gan/XG hydrogel patch, and (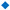) *D. bulbifera* extract/honey-loaded Gan/XG hydrogel patch.

The performances of the hydrogel patches to defend the wound area against contamination from external bacteria were assessed by the infiltration test. Table 5 and Figure 5 show the ability of the hydrogel patches to prevent *S. aureus* and *E. coli* infiltration. Table 5 shows bacterial growths after incubation overnight. The colonies of *S. aureus* and *E. coli* were observed on agar plates representing the positive control, while growths of bacteria on the hydrogel patches were not observed.

As shown in Figure 5a, the turbidity values of broth incubated with agar under all hydrogel patches at 4 h were lower than those incubated with agar containing *S. aureus* and were not significantly different from the control. Moreover, all hydrogel patches clearly inhibited *E. coli* infiltration from external sources (Figure 5b). After incubation in broth for 4 h, the turbidity values of the broth incubated with the agar under loaded and unloaded hydrogels were not significantly different from the control. Broth incubated with the positive control showed time-dependent proliferation.

### 3.9. Antioxidant Activity

**Figure 6:**
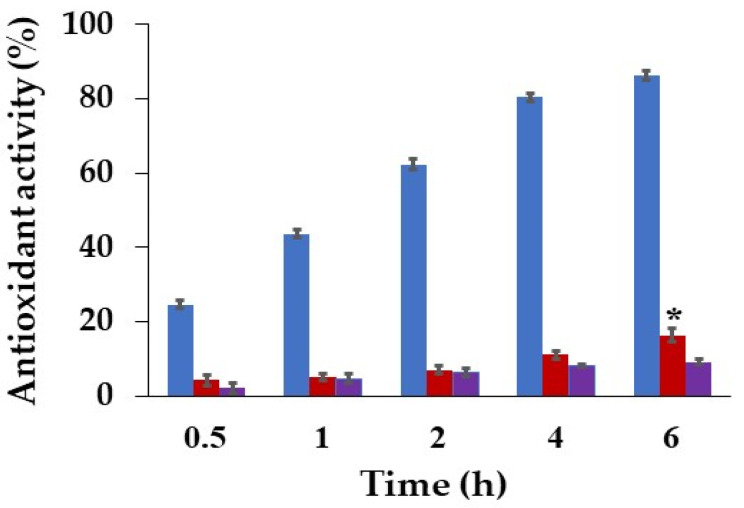
Antioxidant activities of (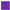) Gan/XG hydrogel patches, (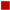) honey-loaded Gan/XG hydrogel patches, and (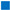) *D. bulbifera* extract/honey-loaded Gan/XG hydrogel patches at 0.5, 1, 2, 4, and 6 h. * *p*-value < 0.05 of honey-loaded Gan/XG compared with Gan/XG at the same concentration.

The antioxidant activities at 0.5, 1, 2, 4, and 6 h of treatments of the hydrogel patches are shown in Table 6 and Figure 6. The results showed that antioxidant activities of the honey-loaded Gan/XG and the unloaded patch were nearly the same after being incubated for 0.5, 1, and 2 h. In addition, the honey-loaded hydrogel patch exhibited significantly higher antioxidant properties compared with the unloaded patch after being incubated for 6 h, which was similar to the antioxidant activity of the previously described honey-loaded dressing [32,33]. The antioxidant ability improved when the incubation time was increased due to the release of honey from the hydrogel patch. Release of honey from honey-loaded Gan/XG was complete within 3 h. The release curve is shown in the Supplementary Materials.

The antioxidant activity of the *D. bulbifera* extract/honey-loaded Gan/XG after being incubated for 1 h significantly increased when the incubation time increased. The result of the *D. bulbifera* extract/honey-loaded Gan/XG correlated with a previous study showing that the ethanolic extract of *D. bulbifera* bulbils exhibited antioxidant activity by the DPPH radical scavenging assay [25]. The antioxidant patches are the alternative for treatment of difficult-to-heal wounds because the antioxidants help to manage wound oxidative stress and balance the level of reactive oxygen species [37,38,39].

### 3.10. NHF Cell Viability

**Figure 7:**
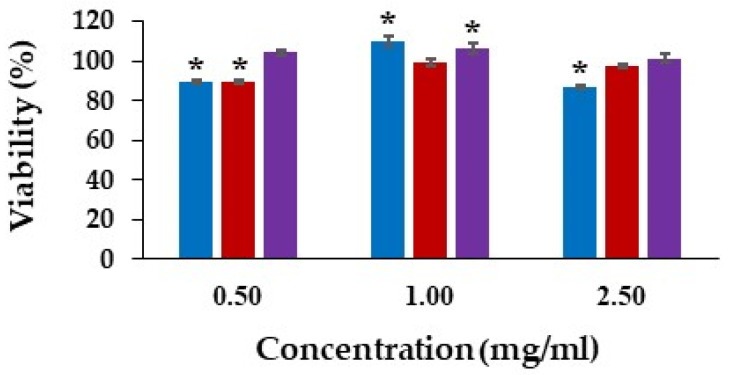
Viability of fibroblast cells after 24 h treated with (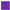) Gan/XG hydrogel patches, (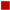) honey-loaded Gan/XG hydrogel patches, and (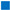) *D. bulbifera* extract/honey-loaded Gan/XG hydrogel patches. * *p*-value < 0.05 compared to untreated cells (control group).

The viability of fibroblast cells exposed with the loaded and unloaded hydrogel patches after a 24 h incubation period was investigated using the MTT assay. Untreated fibroblasts represented the control and were regarded as having 100% viability. The viabilities of all treatment groups with concentrations of 0.5, 1.0, and 2.5 mg/mL higher than 80% were non-toxic to fibroblasts. Moreover, the viability of cells treated with the extraction media of the *D. bulbifera* extract/honey-loaded Gan/XG and the unloaded Gan/XG at the concentration of 1 mg/mL was significantly higher than the control (Figure 7). The results of the *D. bulbifera* extract/honey-loaded patch related to the previous study of *D. bulbifera* ethanolic extract with concentrations from 1 to 100 µg/mL that exhibited low toxicity [25].

### 3.11. NHF Cell Proliferation

**Figure 8:**
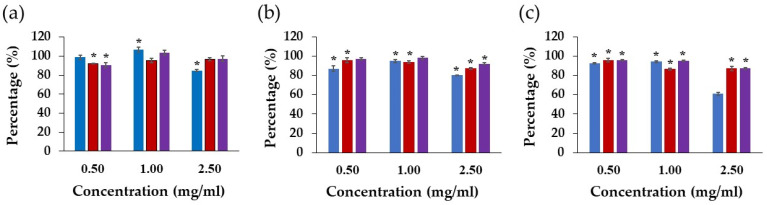
Proliferation of fibroblast cells after (**a**) 24, (**b**) 48, and (**c**) 72 h treated with unloaded (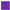) Gan/XG hydrogel patches, (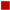) honey-loaded Gan/XG hydrogel patches, and (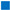) *D. bulbifera* extract/honey-loaded Gan/XG hydrogel patches. * *p*-value < 0.05 compared to untreated cells (control group).

Fibroblast increments after long-term exposure with the extraction media were evaluated by the proliferation assay (Figure 8). The proliferation values of more than 80% were statistically analyzed compared with the control. The honey-loaded Gan/XG and the *D. bulbifera* extract/honey-loaded patch with a concentration of 1 mg/mL increased cell proliferation after incubation for 24 h, whereas only the *D. bulbifera* extract/honey-loaded patch significantly enhanced proliferation compared with the control. Moreover, the *D. bulbifera* extract/honey-loaded patch with a concentration of 2.5 mg/mL inhibited fibroblast proliferation after a long period of incubation.

### 3.12. In Vitro Scratch Assay

The extraction medium at a concentration of 1 mg/mL was chosen for the fibroblast migration test because of its high viability and proliferation effects. Table 7 presents images of the *D. bulbifera* extract/honey-loaded Gan/XG-, the honey-loaded Gan/XG-, and the unloaded Gan/XG-treated fibroblast cells at 0, 24, 48, and 72 h. Untreated fibroblasts were used as a baseline.

**Figure 9:**
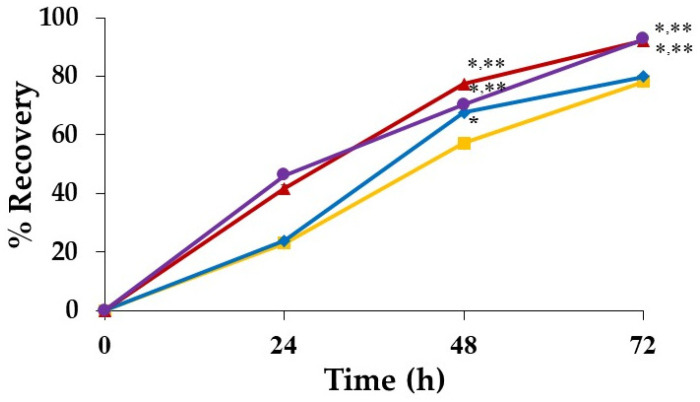
The percentage recovery of the scratched area of (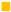) control (untreated cells) and treated cells with extraction medium of hydrogel patches: (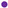) Gan/XG, (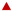) honey-loaded Gan/XG, and (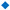) *D. bulbifera* extract/honey-loaded Gan/XG. *p*-value < 0.05 compared to untreated cells (*) and *D. bulbifera* extract/honey-loaded Gan/XG (**) at the same incubation time.

Figure 9 shows the recovery of scratched areas after exposure to the extraction media of hydrogel patches for 24, 48, and 72 h. The gap areas of all groups decreased after 24 h of the incubation period. The scratch areas that were more than 50% after incubation for 48 and 72 h were statistically analyzed. The percentage recovery of the scratched area at 72 h of all samples and the control group were significantly higher than at 48 h. In addition, the percentage recovery at 48 h of all treated groups was significantly different than the untreated cells. Both the honey-loaded Gan/XG hydrogel patches and the unloaded Gan/XG hydrogel patches’ groups could significantly recover the scratched area compared with the untreated group after being incubated for 72 h. The percentage recovery of the cells treated with the honey-loaded Gan/XG and unloaded Gan/XG hydrogel patches was observed to be significantly higher than the *D. bulbifera* extract/honey-loaded Gan/XG hydrogel patches-treated group.

From the migration results, the honey-loaded and the unloaded Gan/XG patches stimulated fibroblast movement within 24 h of incubation. The percentage recovery of the honey-loaded Gan/XG related to an earlier study that explained the migration improvement encouraged by honey [29]. Furthermore, the high percentage recovery of the unloaded hydrogel patch might be due to the high efficiency of XG to stimulate fibroblast migration, as previously reported [13]. The *D. bulbifera* extract/honey-loaded Gan/XG patch significantly enhanced fibroblast movement within 48 h of treatment.

## 4. Conclusions

The optimized film designed and developed using the CCD for hydrogel dressings contained 17.78% *w*/*w* of Gan and 0.1% *w*/*w* of XG. The honey-loaded and the *D. bulbifera* extract/honey-incorporated hydrogel patches were successfully fabricated. Honey loading led to a lower water content but a higher water absorption ability compared with the unloaded patch. The mixture of *D. bulbifera* extract and honey also decreased the water content and increased the water absorption properties of the hydrogel patch; moreover, the water absorption ability was higher than the honey-loaded patches, representing the higher absorption of wound exudate. In addition, mechanical properties of the hydrogel patch, including stiffness, strength, and extensibility, were increased by honey loading. The combination of *D. bulbifera* extract and honey also improved the strength and elasticity of the hydrogel patch. For biological activities, the results of the infiltration test showed that all patches could protect the inside from external bacterial contamination. The antioxidant activities of both loaded hydrogel patches were higher than the unloaded patch. Furthermore, the antioxidant activity of *D. bulbifera* extract was more powerful than honey. The hydrogels exhibited no toxicities to fibroblast cells. The *D. bulbifera* extract/honey-loaded Gan/XG hydrogel enhanced the fibroblast cell proliferation compared with untreated cells after treatment for 24 h. All hydrogel patches improved fibroblast cell migration at 48 h. The unloaded patches and the honey-loaded patches were able to recover the scratch area at 72 h. Therefore, these Gan/XG hydrogel patches could be potential candidates to enhance wound healing.

## Figures and Tables

**Table 1:** The full CCD of the hydrogel patches and measured values (output) of the six responses.

Factors		Responses					
A: Gan (%)	B: XG (%)	Y_1_: Water Absorption (%)	Y_2_: Tensile Strength (MPa)	Y_3_: Young’s Modulus (MPa)	Y_4_: Elongation (%)	Y_5_: Erosion (%)	Y_6_: Water Content (%)
18.62	0.175	505.35	0.184	0.067	284.45	5.44	45.27
18.00	0.100	912.42	0.071	0.015	360.20	26.29	44.01
14.38	0.175	265.64	0.134	0.108	230.60	16.67	49.22
16.50	0.281	247.17	0.405	0.242	210.75	0.00	46.75
15.00	0.250	130.93	0.241	0.176	194.10	6.90	45.52
15.00	0.100	623.50	0.051	0.030	370.15	24.14	50.22
16.50	0.175	532.74	0.290	0.101	273.85	18.33	50.74
16.50	0.175	411.39	0.306	0.125	308.15	25.00	50.91
16.50	0.175	430.18	0.332	0.068	301.65	21.21	50.75
18.00	0.250	269.17	0.302	0.165	246.28	0.00	48.18
16.50	0.175	405.25	0.267	0.099	299.63	24.24	50.81
16.50	0.069	798.35	0.112	0.028	420.15	24.24	46.75
16.50	0.175	439.03	0.327	0.084	255.45	15.42	50.23

**Table 2:** The experimental equations created for various hydrogel patch responses.

Coefficient	Y_1_	Y_2_	Y_3_	Y_4_	Y_5_	Y_6_
b_0_	443.72	0.3043	0.0954	287.75	20.84	50.69
b_1_	95.77	0.0189	−0.0106	14.80	−2.58	−1.14
b_2_	−239.41	0.1044	0.0749	−73.26	−9.73	−0.0660
b_12_	−37.67	0.0101	0.0009	15.53	−2.26	2.22
b_11_	−21.64	−0.0833	−0.0077	−13.56	−4.21	−1.72
b_22_	46.99	−0.0335	0.0160	15.40	−3.67	−1.97
*p*-value	0.0002	0.0001	0.0002	0.0001	0.0018	<0.0001
*R* ^2^	0.9502	0.9565	0.9535	0.9568	0.9062	0.9887

**Table 3:** The physical properties of the hydrogel patches.

Hydrogel Patches	Water Content (%)	Water Absorption (%)	WVTR (g/m^2^/day)
Gan/XG	89.24 ± 0.71	300.41 ± 13.45	N/A
honey-loaded Gan/XG	48.64 ± 0.60	466.89 ± 8.48 *	516.51 ± 12.86
*D. bulbifera* extract/honey-loaded Gan/XG	45.35 ± 1.18	650.19 ± 21.30 *^,^**	576.62 ± 32.52

*p*-value < 0.05 compared to Gan/XG (*) and honey-loaded Gan/XG (**).

**Table 4:** Mechanical properties of the hydrogel patches.

Hydrogel Patches	Young’s Modulus (MPa)	Tensile Strength (MPa)	Elongation (%)
Gan/XG	0.271 ± 0.010	0.146 ± 0.009	59.47 ± 4.14
honey-loaded Gan/XG	0.349 ± 0.022 *	0.229 ± 0.018 *	131.01 ± 7.55 *
*D. bulbifera* extract/honey-loaded Gan/XG	0.253 ± 0.015 **	0.243 ± 0.027 *	191.18 ± 14.88 *^,^**

*p*-value < 0.05 compared to Gan/XG (*) and Honey-loaded Gan/XG (**).

**Table 5:** Infiltration test of the hydrogel patches.

	Gan/XG	Honey-Loaded Gan/XG	*D. bulbifera* Extract/Honey-Loaded Gan/XG
*S. aureus*	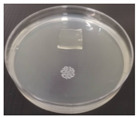	* 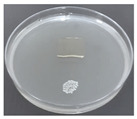 *	* 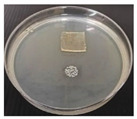 *
*E. coli*	* 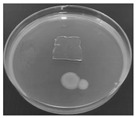 *	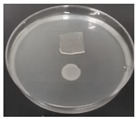	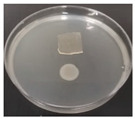

**Table 6:** Antioxidant activity of the hydrogel patches. The left tubes are the control, and the right tubes are the experimental groups.

Samples	Incubation Time (h)			
	1 h	2 h	4 h	6 h
Gan/XG	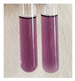	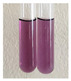	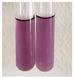	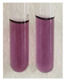
Honey-loaded Gan/XG	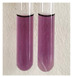	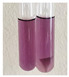	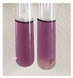	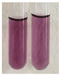
*D. bulbifera* extract/honey-loaded Gan/XG	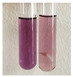	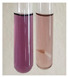	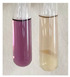	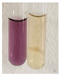

**Table 7:** Migration of fibroblast cells treated with *D. bulbifera* extract/honey-loaded Gan/XG, honey-loaded Gan/XG, and Gan/XG hydrogel patches at 0, 24, 48, and 72 h.

Samples	Migration Test			
	0 h	24 h	48 h	72 h
Control	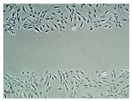	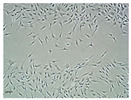	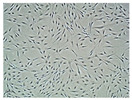	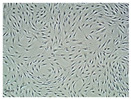
*D. bulbifera* extract/honey-loaded Gan/XG	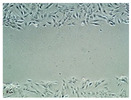	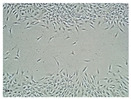	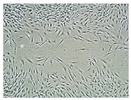	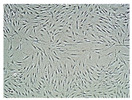
Honey-loaded Gan/XG	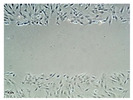	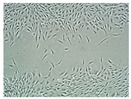	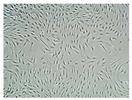	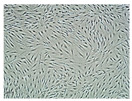
Gan/XG	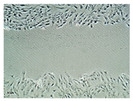	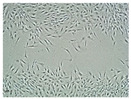	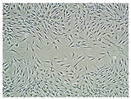	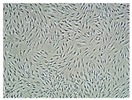

## Data Availability

All data are reported in the article.

## Supplementary Materials

The following supporting information can be downloaded at: https://www.mdpi.com/article/10.3390/pharmaceutics14061302/s1, Reference [40] are cited in the supplementary materials. Figure S1: The honey release curve of honey-loaded Gan/XG patch.
