# Supplementary material for: Formulation and Optimal Design of Dioscorea bulbifera and Honey-Loaded Gantrez®/Xyloglucan Hydrogel as Wound Healing Patches
Source: Pharmaceutics. 2022 Jun 19;14(6):1302. doi: 10.3390/pharmaceutics14061302 (PMC9229440; doi:10.3390/pharmaceutics14061302)
Supplement: Supplementary file 1 [file pharmaceutics-14-01302-s001.zip › pharmaceutics-1752183-supplementary.pdf]

Supporting Information

Honey release behavior

Method

The honey release from hydrogel patches was investigated by reducing sugar measurement. The hydrogel patches were added to 20 mL PBS pH 7.4 and constantly stirred at 37°C in an incubator shaker. The test samples were removed at specific time points (0.5, 1, 1.5, 2, 3, 6, and 9 h) and replaced PBS to complete the initial volume. The amount of honey released in each period was measured using dinitrosalicylic acid reagent (DNS reagent) described previously [1]. Briefly, 1 mL of honey solutions were mixed with 4 mL of DNS reagent. The test samples were heated in boiling water for 5 min and then decreased to room temperature. The absorbance was measured at 540 nm using a spectrophotometer. The percentage of honey release was calculated by the following Equation (S1).

$$\text{Release (\%)} = \frac{A_t}{A_i} \times 100 \qquad \text{(S1)}$$

Where A_i and A_t are amounts of initial honey and release after immersion, respectively.

Result and Discussion

The honey release behavior of the honey-loaded Gan/XG hydrogel patch is shown in Figure S1. Honey was rapidly released from the hydrogels and the complete release was obtained within 3 h. Honey released from the *D. bulbifera* extract/honey-loaded patch could not be evaluated due to the color of extract interfered the measurement. However, honey release from the *D. bulbifera* extract/honey-loaded patch was probably similar to the honey-loaded patch.

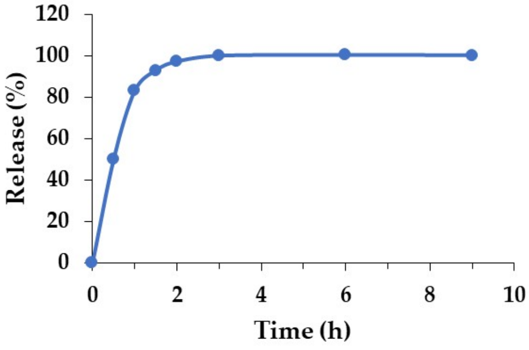

Figure S1. The honey release curve of honey-loaded Gan/XG patch.

Reference

1. Saqib, A.A.N.; Whitney, P.J. Differential behaviour of the dinitrosalicylic acid (DNS) reagent towards mono- and di-saccharide sugars. *Biomass and Bioenergy* **2011**, *35*, 4748-4750.
